# α_2_-Macroglobulin Can Crosslink Multiple *Plasmodium falciparum* Erythrocyte Membrane Protein 1 (PfEMP1) Molecules and May Facilitate Adhesion of Parasitized Erythrocytes

**DOI:** 10.1371/journal.ppat.1005022

**Published:** 2015-07-02

**Authors:** Liz Stevenson, Erik Laursen, Graeme J. Cowan, Betty Bandoh, Lea Barfod, David R. Cavanagh, Gregers R. Andersen, Lars Hviid

**Affiliations:** 1 Centre for Medical Parasitology, Department of Immunology and Microbiology (ISIM), Faculty of Health and Medical Sciences, University of Copenhagen and Department of Infectious Diseases, Copenhagen University Hospital (Rigshospitalet), Copenhagen, Denmark; 2 Institute of Immunology and Infection Research, Center for Immunity, Infection and Evolution, University of Edinburgh, Edinburgh, United Kingdom; 3 Noguchi Memorial Institute for Medical Research, University of Ghana, Legon, Ghana; 4 Department of Molecular Biology and Genetics, University of Aarhus, Aarhus, Denmark; Seattle Biomedical Research Institute, UNITED STATES

## Abstract

Rosetting, the adhesion of *Plasmodium falciparum*-infected erythrocytes to uninfected erythrocytes, involves clonal variants of the parasite protein *P*. *falciparum* erythrocyte membrane protein 1 (PfEMP1) and soluble serum factors. While rosetting is a well-known phenotypic marker of parasites associated with severe malaria, the reason for this association remains unclear, as do the molecular details of the interaction between the infected erythrocyte (IE) and the adhering erythrocytes. Here, we identify for the first time a single serum factor, the abundant serum protease inhibitor α_2_-macroglobulin (α_2_M), which is both required and sufficient for rosetting mediated by the PfEMP1 protein HB3VAR06 and some other rosette-mediating PfEMP1 proteins. We map the α_2_M binding site to the C terminal end of HB3VAR06, and demonstrate that α_2_M can bind at least four HB3VAR06 proteins, plausibly augmenting their combined avidity for host receptors. IgM has previously been identified as a rosette-facilitating soluble factor that acts in a similar way, but it cannot induce rosetting on its own. This is in contrast to α_2_M and probably due to the more limited cross-linking potential of IgM. Nevertheless, we show that IgM works synergistically with α_2_M and markedly lowers the concentration of α_2_M required for rosetting. Finally, HB3VAR06^+^ IEs share the capacity to bind α_2_M with subsets of genotypically distinct *P*. *falciparum* isolates forming rosettes *in vitro* and of patient parasite isolates *ex vivo*. Together, our results are evidence that *P*. *falciparum* parasites exploit α_2_M (and IgM) to expand the repertoire of host receptors available for PfEMP1-mediated IE adhesion, such as the erythrocyte carbohydrate moieties that lead to formation of rosettes. It is likely that this mechanism also affects IE adhesion to receptors on vascular endothelium. The study opens opportunities for broad-ranging immunological interventions targeting the α_2_M—(and IgM-) binding domains of PfEMP1, which would be independent of the host receptor specificity of clinically important PfEMP1 antigens.

## Author Summary

Erythrocytes infected by parasites causing severe *P*. *falciparum* malaria often form rosettes by binding a number of uninfected erythrocytes. Several of the parasite proteins involved are known, whereas the identity of the corresponding host receptor(s) on the surrounding erythrocytes is not. Although formation of rosettes often depends on non-immune IgM also binding to the infected erythrocytes, that does not by itself lead to formation of rosettes. Here, we report that the serum protein α_2_-macroglobulin (α_2_M) is able to induce rosetting in several *in vitro* and *ex vivo* parasite isolates. In contrast to IgM, α_2_M supports rosetting on its own, while presence of IgM markedly lowers the concentration of α_2_M required. These findings are explainable by the ability of α_2_M to crosslink at least four individual PfEMP1 molecules, indicating that the role of α_2_M in rosetting is to align multiple parasite adhesion proteins, thereby increasing their combined avidity for carbohydrate receptors on surrounding erythrocytes. Our study suggests a new mechanism whereby *P*. *falciparum* exploits soluble host proteins to avoid immune destruction, by using them to facilitate adhesion of infected erythrocytes to low-affinity carbohydrate receptors, and points to new strategies to interfere with a major pathogenic mechanism of this devastating parasite.

## Introduction

About 630,000 (0.3%) of the approximately 200 million malaria cases each year are fatal [1]. The majority occur among African children below the age of five years, who die of severe *Plasmodium falciparum* malaria [2]. The particular virulence of *P*. *falciparum* parasites is related to the expression of adhesive proteins on the surface of the erythrocytes they infect, and the *P*. *falciparum* erythrocyte membrane protein 1 (PfEMP1) family appears to be of particular importance in this respect. Each parasite genome encodes about 60 antigenically diverse PfEMP1 proteins composed of a series of Duffy binding-like (DBL) and Cysteine-rich inter-domain region (CIDR) domains. The PfEMP1 proteins are expressed on knob-like protrusions on the infected erythrocyte (IE) surface, where they mediate adhesion of IEs to a range of host endothelial receptors [3–6]. IE sequestration can cause inflammation and organ dysfunction, and can lead to severe and life-threatening complications [7]. In addition to adhesion to receptors in various organs, some *P*. *falciparum*-IEs also have the capacity to bind receptors on uninfected erythrocytes, leading to IE/erythrocyte aggregates called rosettes [8,9]. Although the mechanisms and functional significance of rosetting remain unclear, it has repeatedly been associated with parasites causing severe *P*. *falciparum* malaria [10–12]. Rosetting involves soluble factors in human serum, in apparent contrast to other PfEMP1-mediated IE adhesion to clinically important endothelial protein receptors such as intercellular cell adhesion molecule 1 (ICAM-1) and endothelial protein C receptor [4,6,13]. Several candidates have been proposed (reviewed in ref. [14]), but only pentameric IgM has repeatedly been found to be necessary, although not sufficient, for rosetting to occur [15–17]. The molecular details of this interaction between PfEMP1 and IgM have been described [17,18]. PfEMP1 interaction with the rosetting receptors on surrounding erythrocytes is mediated by N-terminal DBL1 α1.5/6/7/8 domains [19–21], whereas rosette-facilitating IgM binds to the membrane-proximal, C-terminal end of PfEMP1 [17,18]. How IgM can facilitate rosetting despite binding the opposite end of the PfEMP1 molecule facing the erythrocyte receptor is not known. Based on studies of the IgM-binding and rosette-mediating PfEMP1 protein HB3VAR06, we recently proposed that the function of IgM in rosetting could be cross-linking of PfEMP1 molecules [17], thereby overcoming their low individual affinity for their erythrocyte receptors, which are suspected to be mainly sulfated carbohydrate moieties [12,22–24]. However, we found that IgM alone was not sufficient to facilitate HB3VAR06-mediated rosetting, in line with earlier findings [15,16], and we therefore set out to identify the missing serum component(s) involved in rosetting and describe their involvement in the interaction.

## Results

### Identification of the human serum protein α_2_M binding to the PfEMP1 protein HB3VAR06

**Fig 1:**
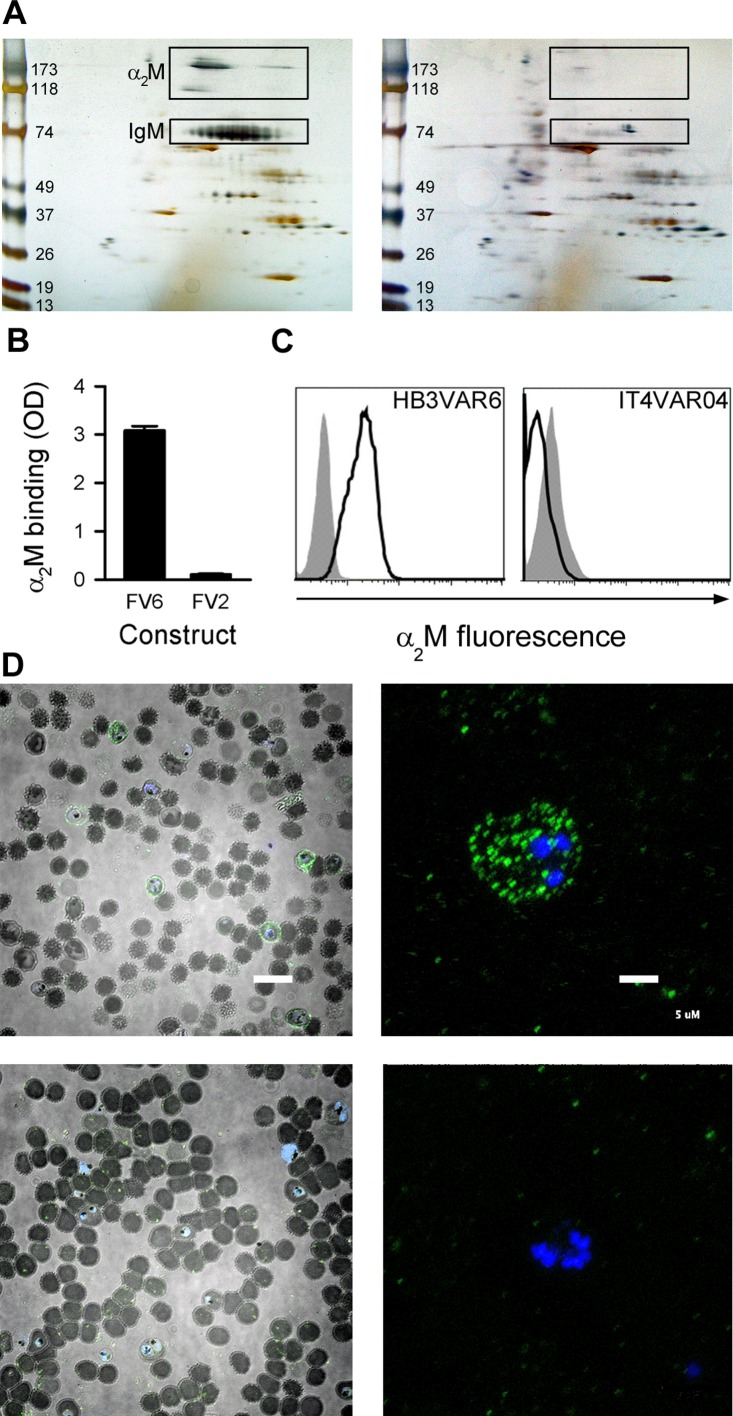
Identification of α_2_M as the soluble serum factor binding HB3VAR06. (A) 2D gel electrophoresis of serum components pulled down with recombinant full-length HB3VAR06 (FV6; left) or PFD1235w-DBLβ3_D5 (right). Spots subsequently identified as α_2_M and IgM in the left panel are boxed. Molecular weight (kDa) markers are shown along the left margins. (B) Binding of α_2_M to recombinant full-length HB3VAR06 (FV6; left) and IT4VAR04 (FV2; right), measured by ELISA. Means and SD are indicated. (C) Binding of α_2_M to HB3VAR06^+^ IEs (left) and IT4VAR04^+^ IEs (right), measured by flow cytometry. Control sample labeling (no α_2_M added) is indicated by gray shading. (D) Fluorescence micrographs of DAPI-labeled HB3VAR06^+^ IEs in the presence (top) and absence (bottom) of fluorescein isothiocyanate-labeled α_2_M at low (scale bar: 20 μm; left) and high (scale bar: 5 μm; right) magnification are shown.

Pentameric IgM facilitates HB3VAR06-mediated rosetting, although additional unidentified serum components are required [17]. To identify these additional components, we used His-tagged recombinant full-length HB3VAR06 (FV6) coupled to magnetic epoxy resin beads to identify serum proteins with affinity for FV6. The proteins isolated by this pull-down technique were separated by 2-dimensional (2D) gel electrophoresis (Fig 1A). Two candidate spots with an apparent molecular weight of 180 kDa and a third candidate spot at 118kDa were detected, in addition to the expected 75-kDa band corresponding to the IgM heavy chain (Fig 1A, left). These spots were not observed in parallel experiments with a recombinant, His-tagged DBL domain (DBLβ3_D5) of a PfEMP1 protein (PFD1235w) not mediating rosetting [25,26] (Fig 1A, right). Matrix-assisted laser desorption/ionization (MALDI) time-of-flight (TOF)/TOF mass spectrometry analysis identified all three candidate spots as monomer subunits of the human protease inhibitor α_2_-macroglobulin (α_2_M). α_2_M is a 720-kDa homotetrameric glycoprotein composed of four disulfide-linked 180-kDa subunits that form a cage-like structure [27], which circulates at physiological concentrations of 3–4 μM (2–3 mg)/mL [28]. Among its many roles in regulation and transport, it is best known as a broad-spectrum protease inhibitor (reviewed in ref. [29]). The observed affinity of α_2_M for HB3VAR06 was confirmed by enzyme-linked immunosorbent assay (ELISA) and flow cytometry, demonstrating binding of α_2_M to FV6 and to native HB3VAR06 on IEs (Fig 1B and 1C). In contrast, α_2_M did not bind to the VAR2CSA-type PfEMP1 IT4VAR04, neither recombinant full-length protein (FV2) nor native IT4VAR04 on IEs (Fig 1B and 1C). Fluorescence microscopy of α_2_M-labeled HB3VAR06^+^ IEs revealed a punctate pattern of fluorescence (Fig 1D, top panels) resembling that characteristic of antibody-labeled PfEMP1 [30,31], whereas parallel samples including the detection antibodies but omitting α_2_M did not produce this pattern (Fig 1D, lower panels).

### HB3VAR06 can bind native α_2_M

**Fig 2:**
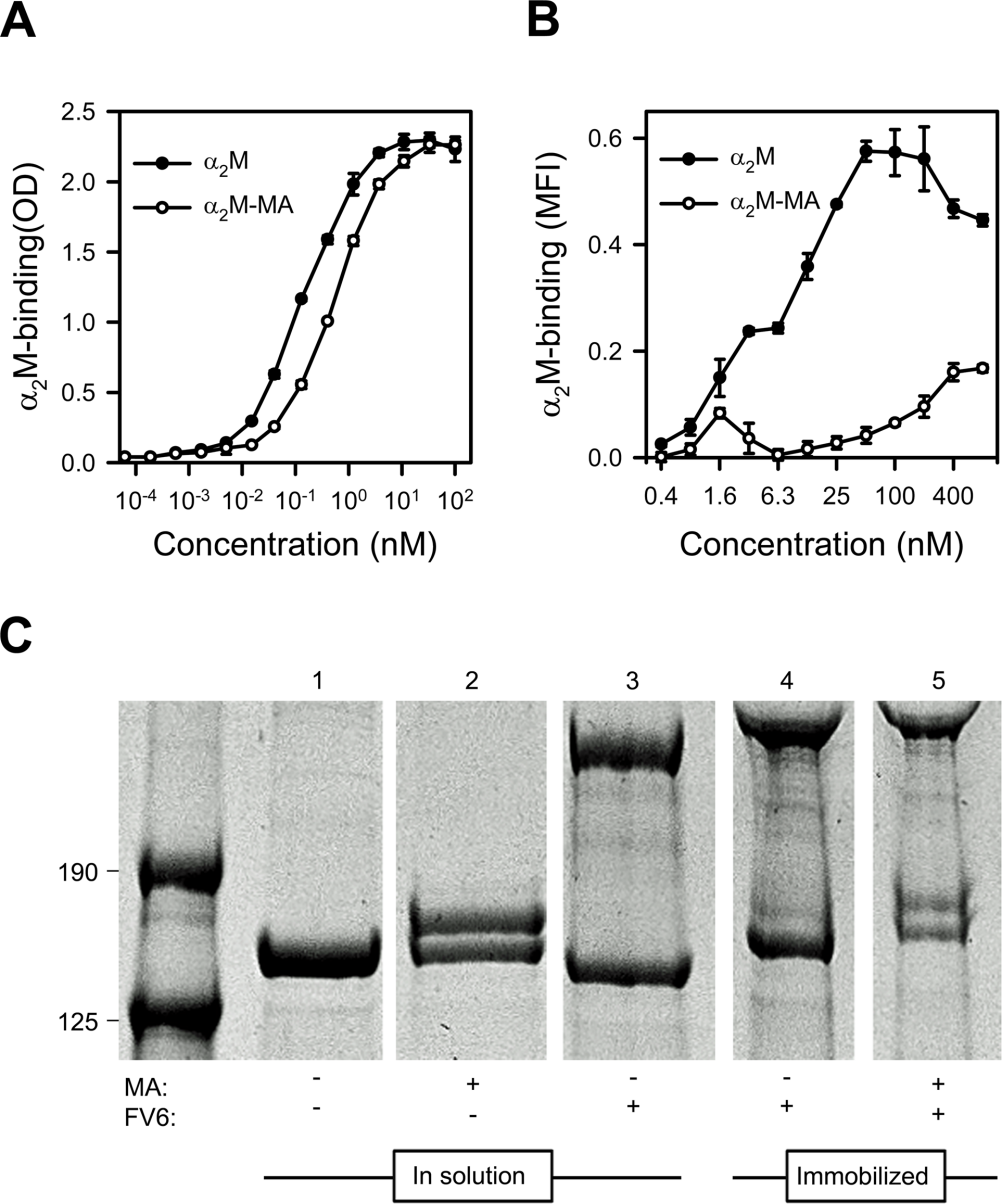
Binding of native and MA-activated α_2_M to HB3VAR06. (A) Titration of binding of native α_2_M (black circles) and α_2_M-MA (white circles) to recombinant full-length HB3VAR06 measured by ELISA. Means and SD are indicated. (B) Titration of binding of native α_2_M (black circles) and α_2_M-MA (white circles) to HB3VAR06^+^ IEs measured by flow cytometry. Means and SD are indicated. (C) Activation of α_2_M measured by SDS gel electrophoresis of soluble and immobilized α_2_M in the presence of mPEG: soluble α_2_M alone (lane 1), soluble α_2_M and MA (lane 2), soluble α_2_M and FV6 (lane 3), bead-immobilized α_2_M-FV6 complexes alone (lane 4), and bead-immobilized α_2_M-FV6 complexes and MA (lane 5). While native α_2_M was detectable in all lanes, activated α_2_M having a higher molecular weight than native α_2_M due to incorporation of mPEG was only detected in the presence of MA (lanes 2 and 5).

Circulating native α_2_M has a serum half-life of several hours [32]. It contains four “bait” regions that are highly susceptible to proteases, and an internal thioester that is sensitive to cleavage by small nucleophiles such as methylamine (MA). Cleavage of either the “bait” regions or the thioester results in a Venus flytrap-like conformational change in α_2_M. This exposes the thioester, which then covalently “captures” the protease in the cage-like structure and exposes the receptor-binding sites in α_2_M. *In vivo*, the serum half-life of this “activated” α_2_M is therefore short (2–4 min), as it is rapidly cleared from circulation when the receptor-binding sites bind to the α_2_M receptor low density lipoprotein receptor-related protein 1 (CD91) [32,33]. The conformation of α_2_M activated either by protease cleavage of the bait region or by nucleophile cleavage of the thioester are structurally and functionally similar and both bind CD91 (reviewed in ref. [34]). We therefore used native α_2_M and MA-activated α_2_M (α_2_M-MA) to examine which of the two conformational states could bind HB3VAR06. ELISA analysis showed that FV6 bound approximately four times more native α_2_M than α_2_M-MA (Fig 2A). Corresponding flow cytometry data showed concentration-dependent binding of native α_2_M binding to HB3VAR06^+^ IEs, whereas α_2_M-MA did not bind well to IEs, even at high concentrations (Fig 2B).

α_2_M is a promiscuous protein with about one hundred binding partners listed in the BioGRID database [35], and in most cases these interactions trigger activation of α_2_M. Methyl-poly(ethylene glycol) maleimide (mPEG) forms a covalent bond with the thiol group liberated following α_2_M activation. This can be detected by denaturing SDS gel electrophoresis as an increase in the molecular weight of activated α_2_M. We used this system to test whether interaction with HB3VAR06 activated α_2_M. In solution and in the presence of mPEG, MA activated native α_2_M as expected (Fig 2C, lane 2), whereas FV6 did not (lane 3). Immobilization of FV6-bound α_2_M to epoxy beads did not cause activation of α_2_M (lane 4), but the α_2_M in the immobilized complex could be activated by MA (lane 5).

### α_2_M binds the penultimate C-terminal DBLξ2 domain of HB3VAR06

**Fig 3:**
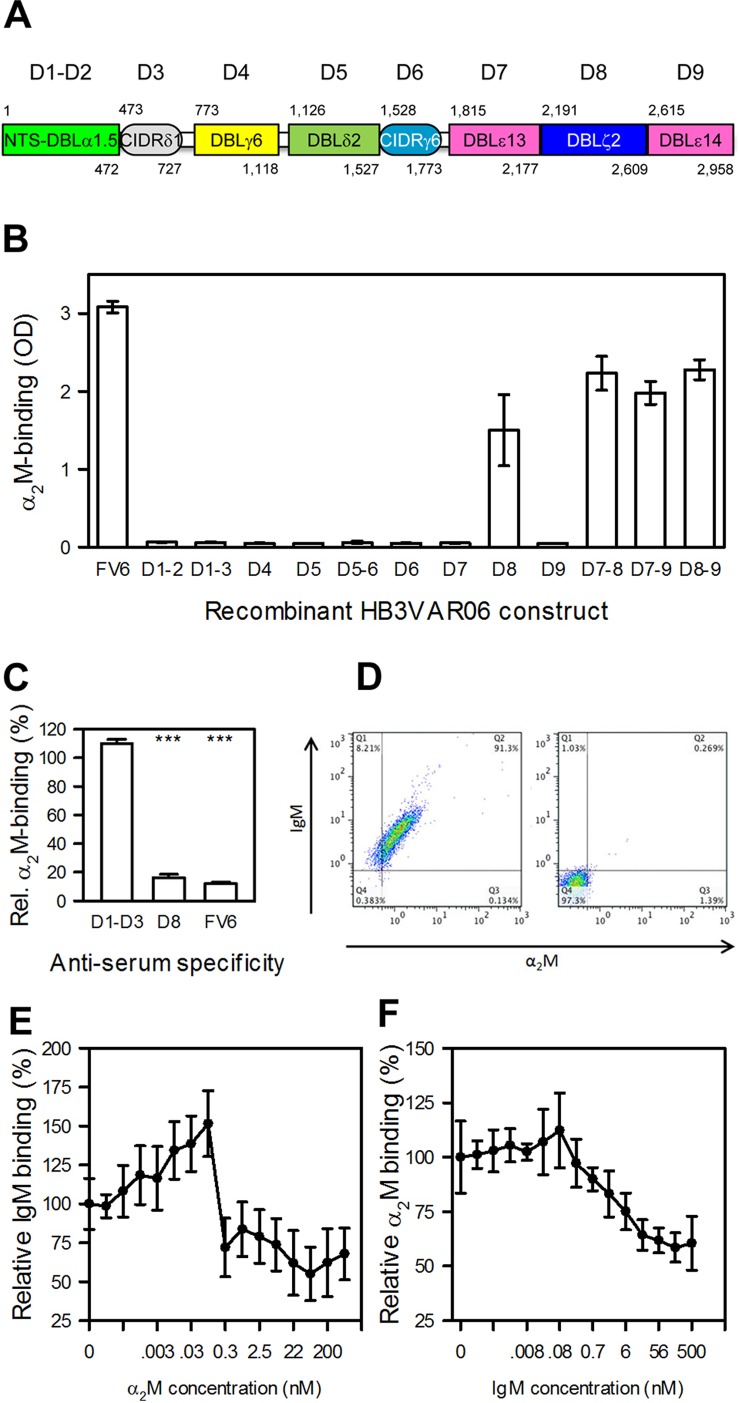
Identification and characterization of the α_2_M-binding domain in HB3VAR06. (A) Schematic representation of the domain structure of HB3VAR06. Domain nomenclature as described by Rask *et al*. [37], as well as the first and last amino acid in each domain are indicated. (B) Binding of α_2_M to recombinant HB3VAR06 single-, double-, and triple-domain constructs (labeled as in panel A) as well as to full-length HB3VAR06 (FV6) measured by ELISA. Means and SD are indicated. (C) Inhibition of α_2_M binding to HB3VAR06^+^ IEs by anti-sera raised against the N-terminal head structure (D1–D3), DBLξ2 (D8), and full-length HB3VAR06 (FV6), respectively, measured by flow cytometry. Means and SD relative to binding without anti-serum are indicated. (D) Simultaneous labeling of HB3VAR06^+^ IEs by α_2_M and IgM (left), measured by flow cytometry. A control experiment with all detecting antibodies present but without α_2_M and IgM is shown to the right. (E) Inhibition of IgM binding to HB3VAR06^+^ IEs by increasing concentrations of α_2_M, measured by flow cytometry. Means and SD relative to binding in the absence of α_2_M are indicated. (F) Inhibition of α_2_M binding to HB3VAR06^+^ IEs by increasing concentrations of IgM, measured by flow cytometry. Means and SD relative to binding in the absence of IgM are indicated.

HB3VAR06 is composed of an N-terminal segment followed by eight DBL and CIDR domains (Fig 3A). In an earlier study, we used recombinant DBL domain constructs from HB3VAR06 to show that IgM binds to the penultimate DBL domain (D8; DBLξ2) near the C-terminus [17]. Taking a similar approach here, we could show that α_2_M also bound exclusively to recombinant single-, double-, and triple-domain constructs containing DBLξ2 (Fig 3B). False signals due to contaminating IgM in the primary or secondary antibodies used to detect α_2_M were ruled out in control experiments without α_2_M (S1 Fig).

Flow cytometry analysis of HB3VAR06^+^ IEs showed that FV6- and DBLξ2-specific antisera significantly reduced α_2_M binding, whereas an NTS-DBLα-CIDRδ-specific anti-serum did not (Fig 3C). Thus, IgM and α_2_M bound to the same domain in HB3VAR06. IgM and α_2_M are high-molecular weight proteins (about 900 kD and 720 kD, respectively), but either could bind HB3VAR06^+^ IEs in the presence of the other (Fig 3D). In competitive binding experiments, each protein reduced the binding of the other by approximately two-fold (Fig 3E and 3F). The results indicate that IgM and α_2_M bind to separate HB3VAR06 molecules with similar affinities, although we cannot rule out that individual HB3VAR06 molecules might be able to accommodate both IgM and α_2_M simultaneously.

### α_2_M is required for HB3VAR06-mediated rosetting and acts synergistically with IgM

**Fig 4:**
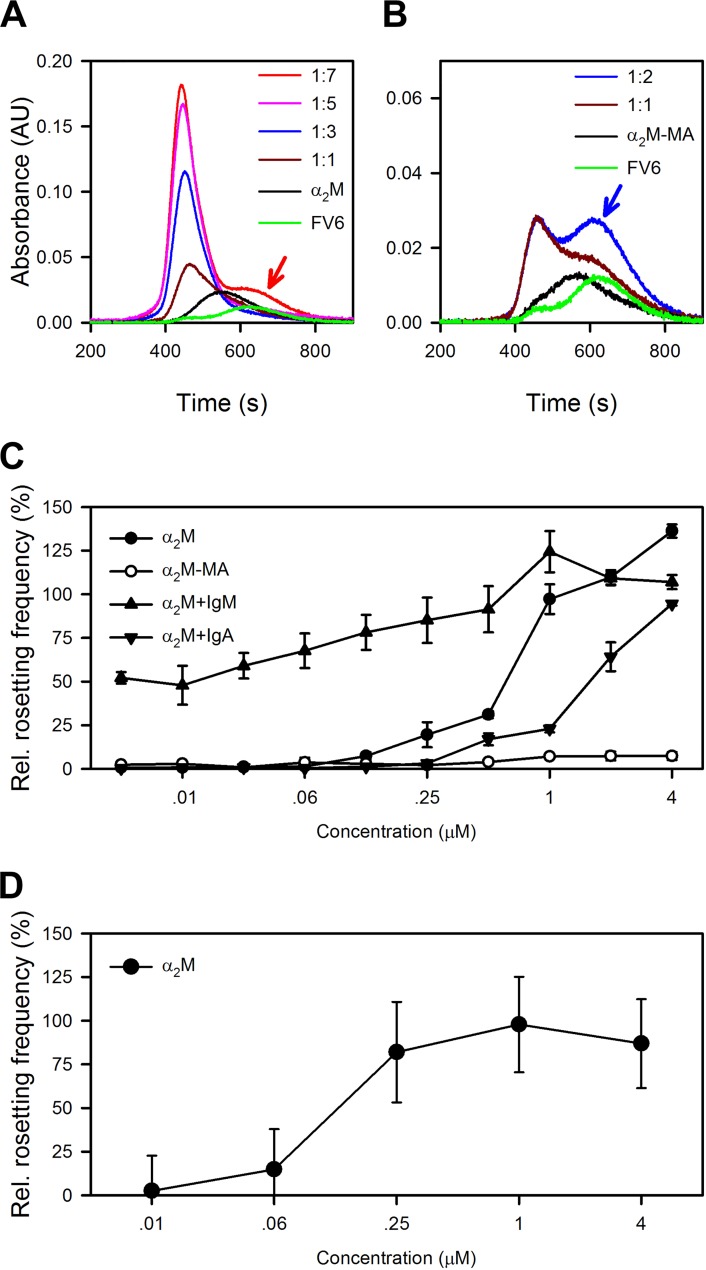
Capacity of α_2_M to induce resetting. (A) Size-exclusion chromatography of α_2_M alone (black), recombinant full-length HB3VAR06 alone (FV6; green), or the two together at α_2_M:FV6 molar ratios of 1:1 (brown), 1:3 (blue), 1:5 (pink), and 1:7 (red), measured by size-exclusion chromatography. The prominent shoulder of unbound FV6 at 1:7 is indicated by a red arrow. (B) Size-exclusion chromatography of α_2_M-MA alone (black), recombinant full-length HB3VAR06 alone (FV6; green), or the two together at α_2_M-MA:FV6 molar ratios of 1:1 (brown) and 1:2 (blue), measured by size-exclusion chromatography. The prominent shoulder of unbound FV6 at 1:2 is indicated by a blue arrow. (C) Rosetting of HB3VAR06^+^ IEs in Albumax medium at different concentrations of α_2_M (black circles), α_2_M-MA (○), and α_2_M in the presence of fixed concentration (3 mg/mL) IgM (black point-up triangles) or IgA (black point-down triangles). Means and SDs relative to rosetting in serum-containing medium are indicated. (D) Ability of α_2_M to restore the capacity of IgM-depleted serum to support rosetting of HB3VAR06^+^ IEs. Means and SD are indicated.

Size-exclusion chromatography analysis showed that saturation of native α_2_M by FV6 occurred at a α_2_M:FV6 ratio of at least 1:4, whereas saturation of α_2_M-MA binding to FV6 was reached already at a 1:1 ratio (Fig 4A and 4B). In the absence of serum, HB3VAR06^+^ IEs do not form rosettes [17], but native α_2_M alone could support rosette formation in serum-free medium in a concentration-dependent manner (Fig 4C). In contrast, α_2_M-MA had no effect on rosetting (Fig 4C). This supports our hypothesis that HB3VAR06 rosetting requires cross-linking of multiple PfEMP1 molecules to overcome the low affinity for receptors on erythrocytes [17], although it may simply reflect the inability of α_2_M-MA to bind to IEs (Fig 2B). Furthermore, the higher multimerization potential of α_2_M than IgM was consistent with our observation that α_2_M could induce rosetting in the absence of other serum factors (Fig 4C), in contrast to IgM [17]. IgM lowered the concentration of α_2_M required for rosetting, indicating that the two serum factors can act in synergy (Fig 4C). This was supported by the observation that rosetting rates were higher when α_2_M and IgM were added together at equimolar concentrations than the theoretical rates calculated as the sum of rosetting rates in medium with either component alone (S2 Fig). In our earlier study [17], we found that IgM-depleted serum did not support rosetting although α_2_M was not intentionally removed. However, addition of exogenous native α_2_M at concentrations lower than those required in serum-free medium restored the ability of IgM-depleted serum to support rosetting (Fig 4D). Thus, endogenous α_2_M levels in IgM-depleted serum appeared to be too low to sustain rosetting, but sufficient to augment IgM-dependent rosetting, when subsequently added.

### α_2_M-binding is not restricted to HB3VAR06

**Fig 5:**
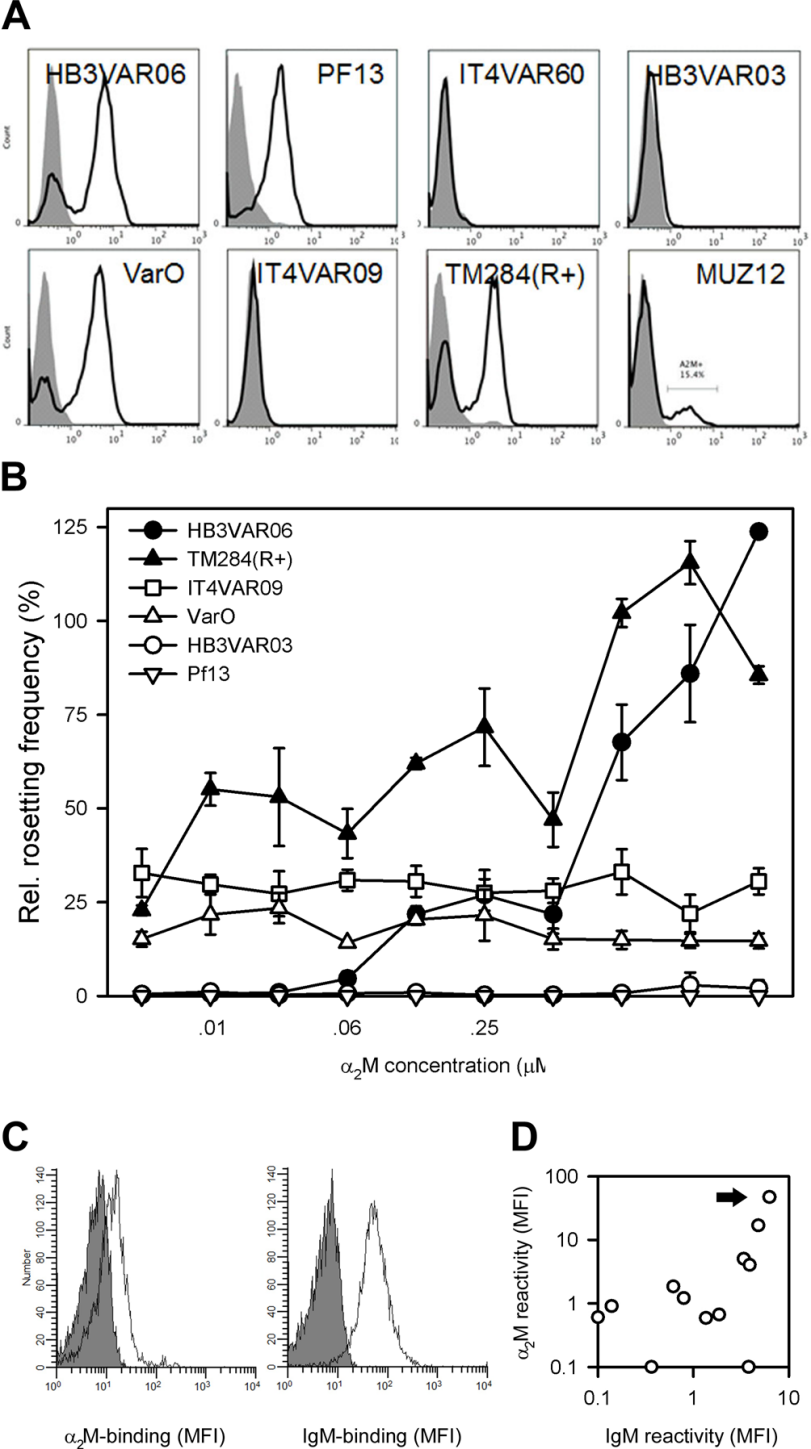
α_2_M binding in parasites not expressing HB3VAR06. (A) Binding of α_2_M to erythrocytes infected by eight genotypically and/or phenotypically different parasite lines, measured by flow cytometry. Control sample labeling (secondary antibody only) is indicated by gray shading. (B) Rosetting frequencies of erythrocytes infected by six genotypically or phenotypically different parasite lines at increasing concentrations of α_2_M but without serum, measured by flow cytometry. Means and SD relative to rosetting in the presence of serum are indicated. (C) *Ex vivo* binding of α_2_M (left) and IgM (right) to erythrocytes infected by a *P*. *falciparum* patient isolate (P25). (D) Correlation of *ex vivo* binding of α_2_M and IgM to erythrocytes infected by *P*. *falciparum* parasites from 12 patients with uncomplicated malaria. Isolate P25 shown in panel C is indicated by an arrow.

Late-stage erythrocytes infected with five of eight laboratory lines selected to express different rosette-mediating PfEMP1 variants contained sub-populations binding α_2_M, evaluated by flow cytometry (Fig 5A). α_2_M induced serum-free rosetting in one (TM284VAR1(R+)) of three α_2_M-binding lines tested, but not in the two others (PF13 and VarO) (Fig 5B). PF13 IEs express the PfEMP1 protein PF13_0003, which does not contain a DBLξ domain with potential to bind IgM, and this parasite did not form any rosettes when grown in serum-free Albumax medium. VarO^+^ IEs have previously been reported to require at least 5% serum to form rosettes [36], but in our hands this parasite line rosetted at low levels (~10%) under serum-free conditions. Although the penultimate C-terminal DBL domain in VarO is a DBLξ domain according to the classification algorithms employed by Rask *et al*. [37] used here, not all such domains support Fc-mediated IgM binding (Jeppesen *et al*., submitted for publication). For both PF13 and VarO, it is tempting to speculate that the inefficiency of α_2_M to induce rosetting in these two parasites is related to absence of synergy between α_2_M and IgM. As expected, α_2_M had no effect on rosetting rates in two lines (HB3VAR03 and IT4VAR09) not binding α_2_M. *Ex vivo* analysis of IEs from 12 Ghanaian children with *P*. *falciparum* malaria showed α_2_M binding in four and IgM binding in five (Fig 5C and 5D). The two phenotypes were highly correlated (P(r = 0.75) <0.005), and IEs from all the α_2_M-binding isolates also bound IgM (Fig 5D). Four children were clinically categorized as suffering from severe *P*. *falciparum* malaria (two had multiple convulsions, one was prostrate, one had respiratory distress). There were no statistically significant associations between clinical presentation (including parasitemia at admission) and either α_2_M- or IgM-binding phenotype (P(r) >0.4 for all). However, our study was neither designed nor powered to thoroughly investigate whether these phenotypes are preferentially found among erythrocytes infected by parasites obtained from patients with severe disease. Together, these data show that the ability to bind α_2_M is a common PfEMP1 phenotype, rather than restricted to HB3VAR06.

## Discussion

Serum factors are required for PfEMP1-mediated rosetting to occur in the majority of genetically and phenotypically distinct *P*. *falciparum* isolates (reviewed in ref. [14]). Pentameric IgM has been a recurring candidate [38,39], and we recently proposed that its role in rosetting is linked to its ability to bind multiple PfEMP1 proteins [17]. However, pentameric IgM only accommodates two HB3VAR06 proteins per IgM molecule, limiting its multimerization potential, and our results showed that additional unidentified serum component(s) are indeed needed for HB3VAR06-mediated rosetting to occur. We therefore set out to close this gap in understanding, and we report here that the unknown serum factor is the abundant protease inhibitor α_2_M. This is the first report that α_2_M plays a role in the pathogenesis of *P*. *falciparum* malaria, and the first demonstration of a single, soluble serum component that is both necessary and sufficient for rosetting of *P*. *falciparum*-IEs.

Antigen-specific pull-down and mass-spectrometry showed that α_2_M bound HB3VAR06, and this finding was confirmed by ELISA and flow cytometry employing recombinant and native HB3VAR06, respectively (Fig 1). We next showed that native α_2_M bound HB3VAR06 much better than activated α_2_M, that this binding did not lead to activation of α_2_M, and that native α_2_M retained its capacity for activation after binding to PfEMP1 (Fig 2). This is similar to what has previously been observed in human-pathogenic Group G Streptococci [40,41], where it has been proposed that surface-bound native α_2_M can protect the bacteria from host protease attack [42]. It is possible that α_2_M-binding to PfEMP1 might provide IEs with a similar type of protection, but this is presently an unconfirmed hypothesis.

α_2_M bound to DBLξ2 near the C-terminus of HB3VAR06, which anchors it in the IE membrane (Fig 3). This is the same domain previously shown to interact with the F_c_ part of IgM [17]. We previously proposed that IgM facilitates rosetting by aligning or “cross-linking” multiple PfEMP1 molecules to increase their combined avidity for receptors on the surrounding erythrocytes, because we found that IgM could bind up to two HB3VAR06 molecules [17]. This “cross-linking” hypothesis is markedly strengthened by the findings reported here. While native α_2_M (which binds at least four PfEMP1 molecules; Fig 4A) supports rosetting on its own, our data also show that α_2_M and IgM act synergistically in facilitating rosetting (Fig 4C and 4D). Thus, IgM allows α_2_M-dependent rosetting to occur at much lower concentrations of α_2_M than would otherwise be required, although IgM on its own does not support rosetting [17]. Activated α_2_M (which cannot bind multiple PfEMP1 molecules; Fig 4B) appears to play no role in rosetting (Fig 4C). Finally, our results showed that α_2_M binding is not restricted to HB3VAR06, as several other genotypically distinct *P*. *falciparum* laboratory lines expressing a variety of PfEMP1 proteins on the IE surface could be labeled by α_2_M (Fig 5A), and that this in some cases led to rosetting (Fig 5B). Furthermore, α_2_M bound to the surface of IEs from Ghanaian *P*. *falciparum* malaria patients (Fig 5C), and the ability to bind α_2_M and IgM were linked phenotypes (Fig 5D). Rosetting has been linked to parasites causing severe malaria in several studies [10,12,43], but in our small data set we did not find significant correlation between α_2_M-binding and severe malaria as defined by the WHO criteria [44]. Future studies are therefore required to determine whether the α_2_M-binding rate is higher among parasites isolated from severe malaria patients, and whether it is associated with particular clinical syndromes and/or expression of particular PfEMP1 proteins.

In this paper we have provided comprehensive evidence that α_2_M is an important component in PfEMP1-mediated rosetting. While the requirement for soluble serum factors in rosetting has long been recognized, their role has not been fully understood. An early study [38], predating the discovery of PfEMP1, suggested that soluble serum factors can form bridges between the IE and the erythrocytes bound to it in the rosette. While our evidence does not formally rule out that possibility, it suggests an alternative mechanism where the soluble serum proteins bind multiple PfEMP1 proteins at their C-terminus, further organizing the knob-specific display of PfEMP1 on the IE surface. The putative α_2_M—(and IgM-) mediated cross-linking of PfEMP1 might improve IE binding avidity by refining the overall topological organization of PfEMP1 or by facilitating intermolecular dimerization of constituent DBL domains. With respect to the former possibility, it is known that the topological and spatial organization of both mannose binding lectin (MBL_3_ oligomers) and its ligand (mannose) contribute to the strength of their adhesive interaction [45]. With respect to the latter, closer packing of multiple PfEMP1 head structures might allow intermolecular dimerization of N-terminal DBL domains. DBL dimerization has been proposed to be “conserved in DBL-domain receptor engagement” and important for the adhesive interaction of DBL domains and their receptors [46,47]. Evidence that the binding sites for Blood Group A antigen and heparin are situated on opposing sides of the head structure of the PfEMP1 protein VarO is compatible with this model, as soluble heparin disrupts VarO-dependent rosetting [36]. However, experimental validation of either hypothesis will require further investigation.

Whether α_2_M binding to PfEMP1 arose to subvert a host immune response mechanism, as might be suggested by the protective function of the complement factor C3 homolog TEP1 in mosquito immunity to these parasites [48], or conversely evolved as a parasite immune-evasive strategy similar to that of Group G Streptococci [40,41] is another question that will require further investigation. In either case, it is plausible that the net result is an increased repertoire of host receptors available for IE adhesion. This is likely to enhance IE retention in tissues, either directly (by enabling IE adhesion to low-affinity vascular receptors, e.g., endothelial Blood Group A antigen [49]) or indirectly (through retention of rosetting IEs in the microvasculature). Either way, this would help the *P*. *falciparum* parasites to avoid destruction in the spleen. We propose that this at least partially explains the correlation between rosetting and severe malaria. Since the proposed α_2_M-mediated cross-linking of PfEMP1 does not depend on the host receptor specificity of the PfEMP1 ligands involved, our finding may open new avenues for PfEMP1-based immune intervention against IE adhesion that target α_2_M/IgM-binding rather than host receptor-binding epitopes. Such anti-adhesive intervention would have a broader scope than has been possible to date.

## Materials and Methods

### Ethics statement

The collection of human plasma samples was approved by the Institutional Review Board of Noguchi Memorial Institute for Medical Research, University of Ghana (Study Number 038/10-11), and by the Regional Research Ethics Committees, Capital Region of Denmark (Protocol H-4-2013-083). All donors were adults and provided written informed consent. All the animal experiments were conducted according to Danish Law and approved (permit 2012-15-2934-00567) by the Danish Animal Procedures Committee (“Dyreforsøgstilsynet”).

### Recombinant parasite proteins, animal anti-sera, human serum, and α_2_M

All recombinant parasite proteins were cloned, expressed, and purified as previously described [50]. In brief, the ectodomains of HB3VAR06, IT4VAR60 (Met_1_ to Ser_2,136_) and IT4VAR09 (Met_1_ to Cys_2,345_) were codon-optimized for insect cell expression by GeneArt (Regensburg, Germany). Full-length, single-, double-, and triple-domain constructs were cloned into the pAcGP67-A vector (BD biosciences, San Jose, CA), transfected and amplified in Sf9 insect cells before being purified from the supernatant of High-Five insect cells via affinity chromatography on HisTrap HP columns (GE Healthcare, Fairland, CT). Recombinant proteins representing PFD1235w-DBLβ3_D5 and the full-length VAR2CSA-type PfEMP1 IT4VAR04 (FV2) were expressed as previously described [26,51]. The ICAM-1-binding full-length PfEMP1 protein IT4VAR13 (FV13) [52] was a kind gift from Thomas Lavstsen. PfEMP1 domain boundaries and sequences not explicitly given above can be obtained from the VarDOM server (http://genome.cbs.dtu.dk/services/VarDom/) [37].

Animal anti-sera specific for recombinant HB3VAR06, IT4VAR09, and IT4VAR60 constructs were raised in rats and rabbits as previously described [17]. All the animal experiments were conducted according to Danish Law and approved by the Danish Animal Procedures Committee (“Dyreforsøgstilsynet”) (permit 2012-15-2934-00567). The mouse monoclonal antibodies specific for the PfEMP1proteins VarO (D1568) and PF13 (J321) were a kind gift from Inès Vigan-Womas and Odile Mercereau-Puijalon. Anti-serum specific for the N-terminal head structure (NTS-DBLα-CIDRα1.4) of HB3VAR03 has been described previously [53] and was a kind gift from Alex Rowe. Human serum was collected and pooled from ten anonymous and healthy blood bank donors without previous exposure to *P*. *falciparum* antigens.

α_2_-macroglobulin (α_2_M) (Sigma) and MA-activated α_2_M (α_2_M-MA; BioMac, Leipzig, Germany) were purchased or extracted from human serum by Zn^2+^-chelate affinity as described [54]. This was followed by gel filtration on a Superdex 200 (GE Healthcare) according to the manufacturer’s instructions. MA-induced conversion of serum-purified native α_2_M to activated α_2_M (α_2_M-MA) in the presence of iodoacetamide was carried out as described before [55].

### Pull-down and identification of α_2_M

FV6 or PFD1235w DBLβ3_D5 (100 μg) were coupled to M-270 epoxy Dynabeads (3 mg; Life technologies, Carlsbad, CA) overnight at 4°C according to manufacturer’s instructions. Following washing (3× in 0.1 M NaPO_4_ buffer containing 0.1% Ig-free bovine serum albumin (BSA;Rockland, USA; pH7.4), the beads were incubated in non-heat-inactivated human serum (500 μL, 1 h, 4°C) on a rotating mixer. Finally, the beads were washed (3× in NaPO_4_ buffer with 0.05% Tween 20) before bound proteins were eluted in citrate (50 μL, 0.01 M, pH 3.1). Eluted products were extensively dialyzed against NaPO_4_ buffer (0.1 M) before the protein concentration was assessed by measurement of absorbance at 280 nm, and 5 μg added to rehydration buffer (8 μL, immobilized pH gradient (IPG) buffer pH 3–10; Life Technologies) with dithioerythritol (0.05 g). The 7 cm IPG strips (Life Technologies) for 2D gel electrophoresis were rehydrated (14 h) before a three-step electrophoresis program was run on an IPGPhor machine (Amersham, Buckinghamshire, UK) as described [56]. Strips were equilibrated and second dimension electrophoresis run using pre-cast 4–12% Bis-Tris Zoom gels according to manufacturer’s instructions (Life Technologies).

Gels were silver stained by sequential incubations in fixation solution (10% C_2_H_4_O_2_, 40% C_2_H_6_O, 30 min), sensitizing solution (30% C_2_H_6_O, 0.2% Na_2_S_2_O_3_, 0.8 M NaOAc, 30 min), dH_2_O (3×5 min), silver stain (0.25% w/v AgNO_3_ solution, 20 min), dH_2_O (2×1 min), developing solution (0.0074% w/v CH_2_O, 0.25 M Na_2_CO_3_, 5 min), and stopping solution (1.46% ethylene-diamine-tetra-acetic acid-Na_2_, 10 min).

### Mass spectrometry

Gel spots of interest were excised and cut into 1 mm^3^ cubes, de-stained by washing in acetonitrile, and subjected to reduction and alkylation before in-gel digestion with trypsin at 37°C using a ProGest Investigator in-gel digestion robot (Digilab, Marlborough, MA) and standard protocols [57]. Digested peptides were extracted with 10% formic acid, and applied (0.5 μL) to the MALDI target along with α-cyano-4-hydroxycinnamic acid matrix (0.5 μL, 10 mg/mL, 50:50 acetonitrile: 0.1% trifluoroacetic acid) and allowed to dry. MALDI-mass spectrometry data were acquired, using a 4800 MALDI TOF/TOF analyzer (ABSciex, Cheshire, UK) equipped with a Nd:YAG 355 nm laser and calibrated using a mixture of peptides. The most intense peptides were selected for mass spectrometry (MS)/MS analysis using GPS Explorer (ABSciex) to interface with the Mascot 2.4 search engine (Matrix Science) and the MS/MS data using Mascot 2.4 directly. Swiss-Prot (Dec 2012) or NCBInr (Aug 2013) databases were interrogated using *Homo sapiens* as species restriction. The data were searched with tolerances of 100 parts-per-million for the precursor ions and 0.5 Da for the fragment ions, trypsin as the cleavage enzyme, assuming up to one missed cleavage, carbamidomethyl modification of cysteines as a fixed modification and methionine oxidation selected as a variable modification.

### *P*. *falciparum* parasites, and *in vitro* culture and selection

*P*. *falciparum* HB3 parasites [58] were maintained *in vitro* and selected for rosetting and expression of HB3VAR06 expression as described [17]. For rosetting laboratory isolates, we used a combination of rosette enrichment (sedimentation in gelatin as described by [59]) and PfEMP1-specific antibody selection [60]. We used rabbit anti-sera raised against the relevant recombinant full-length PfEMP1 proteins to select *P*. *falciparum* IT4 to express IT4VAR60 (such parasites have variously been described in the literature as FCR3S1.2 and PAR+; see [61]) and IT4VAR09 (also known as R29; [62]). *P*. *falciparum* HB3 was selected to express HB3VAR03 using rabbit-antisera against HB3VAR03-NTS-DBLα-CIDRα. *P*. *falciparum* 3D7 expressing PF13_0003 (PF13) and VarO (Genbank: EU9082205) were selected by mouse monoclonal antibodies J321 and D1568 respectively, as described elsewhere [21]. For the isolate TM284var1 (Genbank: JQ684046 and [63]), no PfEMP1-specific anti-sera were available to us. Instead, we used a combination of rosette enrichment and IgM selection. Rosetting in the isolate MUZ12 (Genbank: JQ684048) was maintained by gelatin selection, but no further PfEMP1-specific selection was used. For IgM selection, IEs were incubated in culture medium supplemented with 10% human serum (15 min, rotating plate) before being washed and enriched using rabbit anti-human IgM (Dako, A0426) coupled to Protein A beads (Life technologies). PfEMP1 expression was regularly monitored by flow cytometry using PfEMP1-specific antisera, and only IEs where 60–95% of IEs were specifically labeled were used in the experiments described in this study. All cultures were kept synchronous by twice-weekly sorbitol treatment as described [64]. The genotypic identity and the absence of Mycoplasma infection were verified regularly as described [26]. *P*. *falciparum* IT4 was selected for IE surface expression of the PfEMP1 protein IT4VAR32b by human monoclonal antibody AB01 [65]. IEs from 12 children with *P*. *falciparum* malaria were obtained from venous blood samples collected at Hohoe Municipal Hospital, Hohoe, Ghana. The samples were incubated overnight in candle jars at 37°C, and transported to Accra for analysis by *ex vivo* flow cytometry (see below). These samples were collected with the permission of the Institutional Review Board at Noguchi Memorial Institute for Medical research, University of Ghana (file 026/13-14) and the Ethical Review Committee of the Ghana Health Services (file GHS-ERC 08/05/14).

### Measurements of α_2_M and F_c_-mediated IgM binding to PfEMP1

To assess α_2_M binding by ELISA, 96-well, flat-bottomed MaxiSorp plates (Thermo scientific) were coated with recombinant PfEMP1 constructs (18nM, overnight, 4°C). Following blocking (2 h) in TSM buffer with 1% Ig free BSA, plates were washed and incubated with α_2_M or α_2_M-MA (10 nM). Binding was detected with polyclonal goat-anti-α_2_M (Abcam, Cambridge, UK 7337 1:5,000) or monoclonal mouse-anti-α_2_M (Abcam, 1:1,000), followed by rabbit-anti-goat horseradish peroxidase (1:6,000, Dako, Glostrup, Denmark) or rabbit-anti-mouse horseradish peroxidase (1:2,000, Dako).

Binding of IgM, α_2_M and α_2_M-MA to IEs was detected by flow cytometry, essentially as described [65]. In brief, late-stage IEs (1x10^5^) purified by magnet-activated cell sorting were incubated (30 min, room temperature) with either α_2_M or α_2_M-MA (10 nM). The IEs were washed and incubated first with primary (polyclonal goat-anti-α_2_M, 1:3,000), then fluorescein isothiocyanate-conjugated secondary antibody (polyclonal rabbit-anti-goat, 1:150) (Vector, Peterborough, UK, FI-5000) and ethidium bromide (2 μg/mL). In competition assays, α_2_M, IgM, or HB3VAR06-specific anti-sera (1:20) were added in separate steps. Ethidium bromide labeling was omitted in experiments detecting IgM binding using phycoerythrin-conjugated donkey-anti-human IgM (Jackson ImmunoResearch, Newmarket, UK, 1:400). In assays employing simultaneous surface labeling of IEs by IgM, α_2_M, and HB3VAR06-specific IgG, we used PerCP-conjugated donkey-anti-rabbit antibody (Jackson ImmunoResearch,1:50) to detect HB3VAR06-specific rabbit IgG and Alexa 488-conjugated donkey-anti-goat antibody (Life Technologies, 1:10,000) to detect α_2_M. IE surface labeling was assessed by flow cytometry using a Beckman coulter FC500 instrument (Beckman Coulter, Fullerton, CA) in Copenhagen or a Becton Dickinson FACSCalibur (BD Biosciences, San Jose, CA) in Ghana. List-mode data files were analyzed using FlowJo (v 7.6; Treestar, Ashton, OR) and WinList (v. 6; Verity Software House, Topsham, ME) software.

### Immunofluorescence microscopy

HB3VAR06^+^ IEs (1.2 mL, 5% hematocrit) were pelleted, washed (3×, PBS with 1% Ig-free BSA), and incubated with α_2_M (500 μL, 100 nM, room temperature, 30 min). Following additional washing, the IEs were incubated with polyclonal goat-anti- α_2_M (500 μL, 1:3,000) then Alexa 488-conjugated donkey-anti-goat (Life Technologies, 1:10,000) and DAPI (1 μg/mL). Micrographs of live, unfixed IEs were obtained using a Nikon TE 2000-E confocal microscope equipped with a 60× oil immersion lens (N.A. 1.4) and Nikon EZ-C1 3.5 software (Nikon Instruments, Amsterdam, Netherlands). Images were analyzed using Image J64 software (http://imagej.nih.gov/ij/) [66].

### Detection of α_2_M activation

To test whether binding of α_2_M to FV6 induced thiol-ester conversion of α_2_M to activated α_2_M, FV6 and α_2_M (10 μg) were incubated together in NaPO_4_ buffer (pH 8), in the presence of 0.75 mM mPEG, MW 5000 (mPEG; Laysan Bio, Arab, AL). Incorporation of mPEG was assessed by denaturing SDS-PAGE (5 μL load), comparing activation to that obtained by MA (150 mM; Sigma) in the absence of iodoacetamide. To assess whether α_2_M could be activated after binding to FV6, the FV6: α_2_M complex was immobilized on epoxy beads as described above for the α_2_M pull-down, but using purified α_2_M (200 μg) instead of serum. After removal of unbound α_2_M by thorough washing, FV6-bound α_2_M was incubated with mPEG with or without methylamine as above in NaPO_4_ buffer (pH8, 2 h, 4°C). After additional washing, the beads were incubated with SDS loading buffer and DTT (50 μL), heated (70°C, 5 min) before loading (20 μL) on denaturing SDS gels and processed as above.

### Analytical size-exclusion chromatography

Size-exclusion chromatography (SEC) was performed using a 24-mL Superdex 200 10/30 HR column (GE Healthcare) equilibrated with 50 mM Tris-HCL and 150 mM NaCl (pH 7.4). α_2_M (150 μg) alone and in combination with molar ratios of FV6 were incubated on ice (15 min) and subjected to SEC analysis using a flow rate of 0.5 mL/min and absorbance detection at 280 nm.

### Rosetting assessment

Late-stage IEs (5% parasitemia) were diluted in Albumax medium to 2% hematocrit to achieve a total volume of 20 μL in a 384-well plate. Rosetting agents (α_2_M, α_2_M-MA, IgM, IgA, serum) were concentrated to ≥10 mg/mL and dialyzed against Albumax medium before dilution to the required concentration. The effect of each reagent on IEs (20 μL culture) was tested in triplicate in 384-well plates (20 μL/well, 1 h, 37°C). IEs were labeled with ethidium bromide (2 μg/mL), and rosetting (≥2 erythrocytes bound per IE) assessed by fluorescence microscopy of 200 IEs. In each assay, the rosetting frequency was compared to the positive control (10% serum).

## Supporting Information

S1 Fig Detection of α_2_M-binding to HB3VAR06 is not explainable by presence of IgM in antibody preparations used to label α_2_M. To test whether IgM in the primary, α_2_M-specific, antibody and/or in the HRP-conjugated secondary antibody used to detect α_2_M by ELISA would compromise the assay specificity for detection of α_2_M-binding to HB3VAR06, we coated ELISA plates with FV6 and added goat-anti- α_2_M antibody followed by HRP-conjugated rabbit anti-goat antibody (A, left bar) or mouse-anti- α_2_M antibody followed by HRP-conjugated rabbit anti-mouse antibody (B, left bar). Neither produced any signal, in contrast to the strong signal in control wells coated with α_2_M instead of FV6 (right-hand bars in A and B). Graphs show means and S.D. of triplicates.
(TIF)

S2 Fig Rosetting of HB3VAR06^+^ IEs in serum-free medium supplemented with different concentrations of α_2_M and/or IgM. To verify the synergistic effect of α_2_M and IgM on rosetting, we measured absolute rosetting rates (solid lines) of Albumax-maintained HB3VAR6^+^ IEs after incubation (1 h) in Albumax medium supplemented with IgM alone (black point-up triangles), α_2_M alone (black circles), or equimolar α_2_M and IgM together (black squares) at the concentrations indicated. Synergy was evident, as the observed rosetting rates when α_2_M and IgM were added in combination (black squares) were higher than theoretical rates calculated as the sum of the rates observed in medium containing either α_2_M or IgM (white squares and dashed line). Rosetting rate in medium supplemented with 10% NHS is shown for comparison (right).
(TIF)
