# Supplementary figures and images for: α2-Macroglobulin Can Crosslink Multiple Plasmodium falciparum Erythrocyte Membrane Protein 1 (PfEMP1) Molecules and May Facilitate Adhesion of Parasitized Erythrocytes
Source: PLoS Pathog. 2015 Jul 2;11(7):e1005022. doi: 10.1371/journal.ppat.1005022 (PMC4489720; doi:10.1371/journal.ppat.1005022)

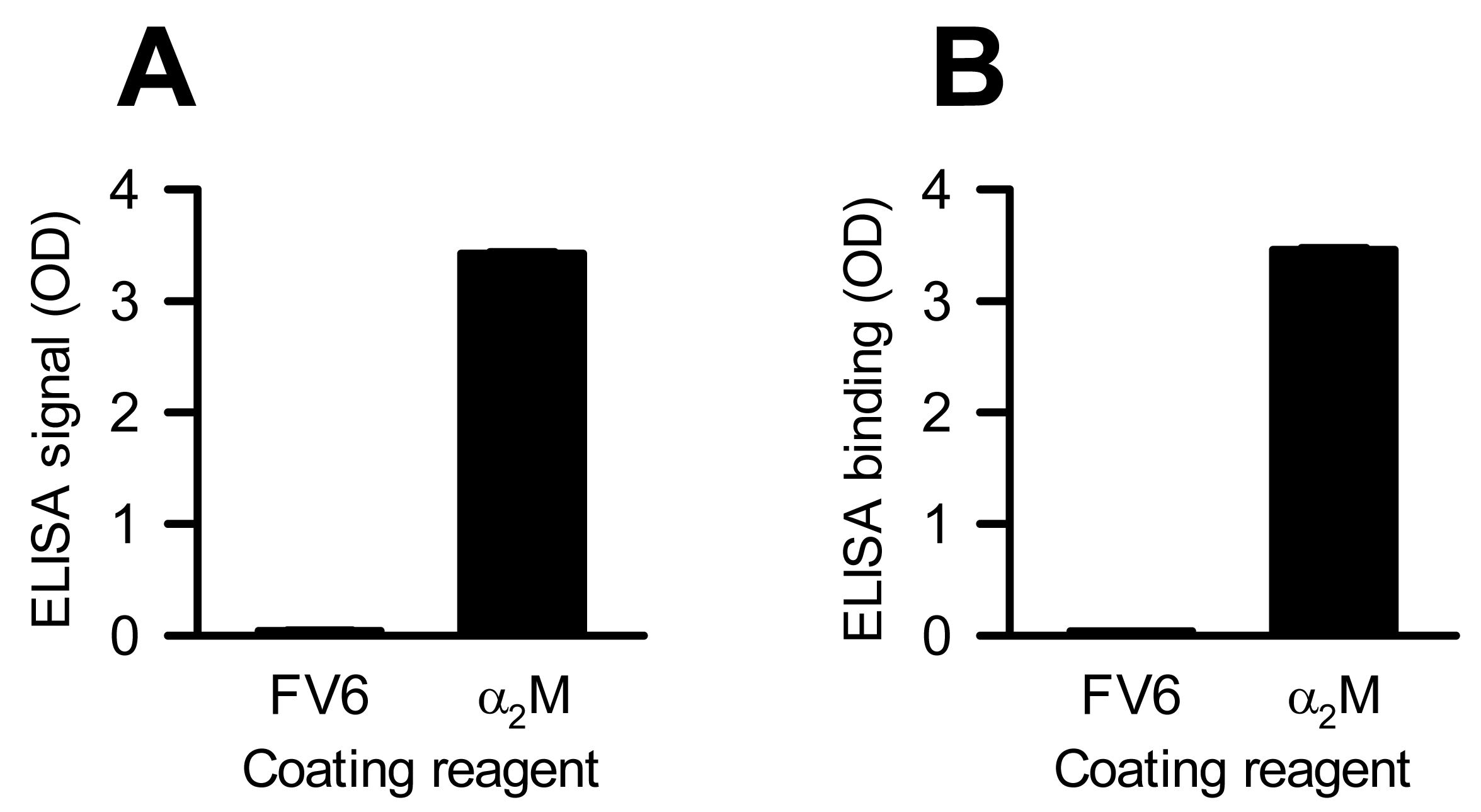

Supplement: S1 Fig — To test whether IgM in the primary, α2M-specific, antibody and/or in the HRP-conjugated secondary antibody used to detect α2M by ELISA would compromise the assay specificity for detection of α2M-binding to HB3VAR06, we coated ELISA plates with FV6 and added goat-anti- α2M antibody followed by HRP-conjugated rabbit anti-goat antibody (A, left bar) or mouse-anti- α2M antibody followed by HRP-conjugated rabbit anti-mouse antibody (B, left bar). Neither produced any signal, in contrast to the strong signal in control wells coated with α2M instead of FV6 (right-hand bars in A and B). Graphs show means and S.D. of triplicates. (TIF) [file ppat.1005022.s001.tif]

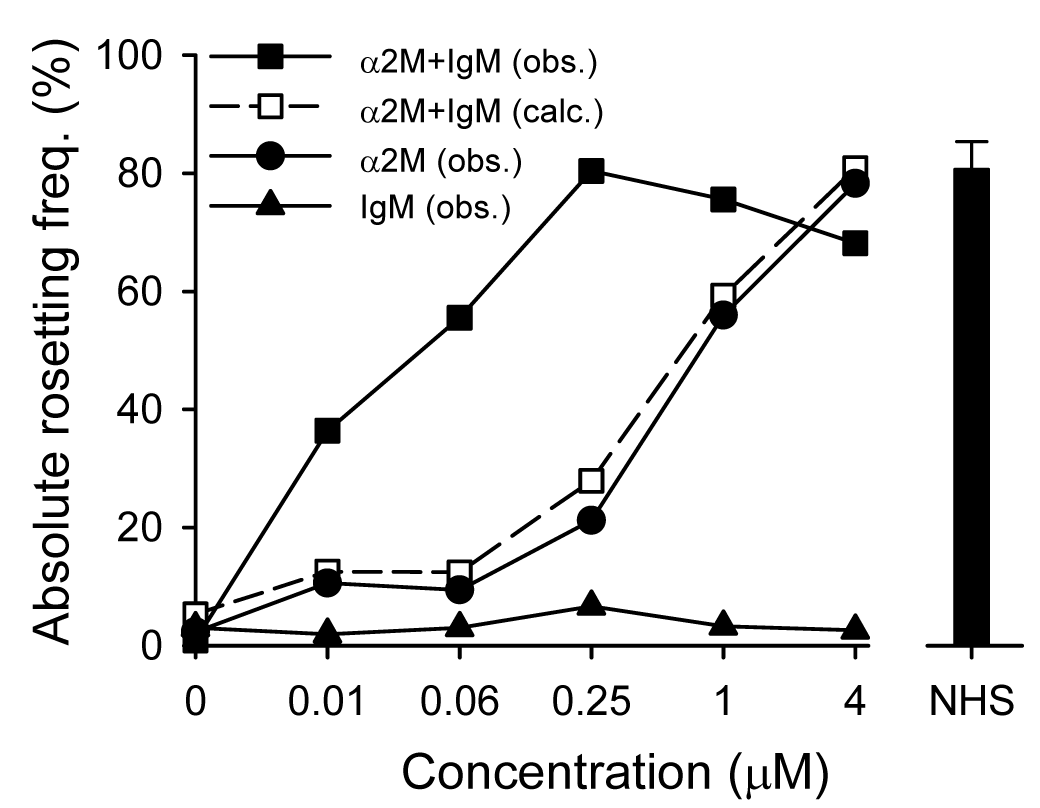

Supplement: S2 Fig — To verify the synergistic effect of α2M and IgM on rosetting, we measured absolute rosetting rates (solid lines) of Albumax-maintained HB3VAR6+ IEs after incubation (1 h) in Albumax medium supplemented with IgM alone (black point-up triangles), α2M alone (black circles), or equimolar α2M and IgM together (black squares) at the concentrations indicated. Synergy was evident, as the observed rosetting rates when α2M and IgM were added in combination (black squares) were higher than theoretical rates calculated as the sum of the rates observed in medium containing either α2M or IgM (white squares and dashed line). Rosetting rate in medium supplemented with 10% NHS is shown for comparison (right). (TIF) [file ppat.1005022.s002.tif]
